# Cu_3_BiS_3_ Nanocrystals as Efficient Nanoplatforms for CT Imaging Guided Photothermal Therapy of Arterial Inflammation

**DOI:** 10.3389/fbioe.2020.00981

**Published:** 2020-08-14

**Authors:** Ran Lu, Jingyi Zhu, Chaowen Yu, Zhonglin Nie, Yong Gao

**Affiliations:** ^1^Department of Vascular Surgery, The First Affiliated Hospital of Bengbu Medical College, Bengbu, China; ^2^School of Pharmaceutical Sciences, Nanjing Tech University, Nanjing, China

**Keywords:** Cu_3_BiS_3_ nanocrystals, atherosclerosis, arterial inflammation, photothermal therapy, CT imaging

## Abstract

Cardio-cerebrovascular diseases caused by chronic inflammatory atherosclerosis seriously damage human health. Nano-photothermal technology has been proven to inhibit the development of vascular inflammation, but the currently reported photothermal agents cannot efficient monitor it during the development of the disease. Herein, we designed and prepared an efficient bifunctional nanoplatform for CT imaging guided photothermal therapy of arterial inflammation. Cu_3_BiS_3_ nanocrystals with a size of about 12 nm were synthesized by a simple hydrothermal method. The as-prepared Cu_3_BiS_3_ nanocrystals showed intense absorption in the NIR region, thus exhibited amazing photothermal effect. The photothermal conversion efficiency of Cu_3_BiS_3_ nanocrystals was reach up to 58.6% under the excitation of an 808 nm laser with a power density of 0.4 W cm^–2^. Cu_3_BiS_3_ nanocrystals can efficiently kill the macrophages both *in vitro* and *in vivo*, which plays an important role in the development of atherosclerosis, thus can be used as an effective way to inhibit the occurrence of hypertension. Importantly, Cu_3_BiS_3_ nanocrystals can be used as an efficient CT contrast agent to monitor carotid inflammation. Our work provides an insight for imaging guided photothermal therapy of arterial inflammation.

## Introduction

Atherosclerosis (AS) is one of the cardiovascular diseases which are the leading cause of human death (Libby et al., 2011; Tzoulaki et al., 2016). Macrophages are the key cells that play an important role in AS formation. Nanoparticle based photothermal therapy (PTT) exhibits a very promising potential due to its less side effects which can kill macrophages resulted from the photothermal effect of nanoparticles, comparing to the traditional whole body chemotherapy, and stent implant surgery having in-stent restenosis risks (Tabas, 2010; Wang et al., 2019). With the development of nanotechnology, PTT technology as an emerging minimally invasive technology shows great potential for the diagnosis and treatment of atherosclerosis (Qin et al., 2015). Photothermal therapy technology is to use photothermal materials with good optical property of near-infrared (NIR) absorption under laser irradiation to achieve local high temperature, thereby effectively killing cells at the lesion site with no surgery (Li et al., 2013, 2017; Song et al., 2017). The NIR (700–1400 nm) laser is an important NIR light source widely used in phototherapy which has very strong penetration ability to biological tissues (Liu et al., 2016). During the progression of atherosclerosis, massive infiltration of monocytes/macrophages and other inflammatory cells and the pathological proliferation of vascular smooth muscle cells promotes plaque formation, secondary stenosis and even occlusion, leading to the occurrence of ischemic diseases (Wang et al., 2019; Zhang et al., 2019). Similar to cancer cells, hyperthermia can also be used to inhibit or kill the aforementioned pathologically expanded cells. Although this minimally invasive technique is widely used in the treatment of cancer, there are few reports on its application to atherosclerosis, and the application of photothermal therapy technology to atherosclerosis has important research significance.

Nano-photothermal technology, especially NIR-laser-driven photothermal therapy technology, has been rapidly developed in recent years. If photothermal treatment technology is to be applied to the treatment of atherosclerosis, the main issue is to explore photothermal agents with low toxicity, multi-function and high photothermal effect (Chen et al., 2013; Li et al., 2014; Shao et al., 2016; Jin et al., 2018). This is consistent with the application of photothermal therapy technology in cancer treatment. Photothermal agents reported to date mainly include carbon materials, organic materials, metal materials and semiconductor materials (Chen et al., 2013). Gold nanostructures are the most extensively and thoroughly studied photothermal agents, but the morphology of noble metal nanostructures will obviously change under the continuous irradiation by lasers which ultimately affect their photothermal properties (Tian et al., 2013). Also, precious metals’ price is also one of the issues to restrict the development of such photothermal agents. Organic compounds mainly include nanoparticles of dyes, polyaniline and polypyrrole (Li et al., 2018; Li and Pu, 2019). This type of photothermal conversion material is biodegradable, but easily photodegradable or photobleached. Carbon photothermal materials, including graphene and carbon nanotubes, have stable performance, but low light absorption coefficient (Robinson et al., 2010, 2011). Semiconductor photothermal nanomaterials show several advantages, such as low price, simple synthesis, high absorption coefficient, stable photothermal performance, and easy functionalization (Meng et al., 2016; Ni et al., 2017; Tan et al., 2017).

Ternary semiconductor nanomaterials can inherit the properties of the corresponding binary semiconductors and produce new characteristics (Liu et al., 2016; Zhou et al., 2016; Li et al., 2017). In particular, copper-based bimetallic sulfides containing elements with imaging properties (such as copper iron sulfur, copper cobalt sulfur, copper manganese sulfur, and copper bismuth sulfur) can theoretically be used as both photothermal agents and imaging contrast agents. Moreover, since the valence of copper in the bimetallic copper-based sulfide is mainly monovalent, it is easily oxidized to divalent and thus degraded in the body (Liu et al., 2016). Therefore, the bimetallic copper-based sulfide is expected to be an ideal photothermal diagnostic agent. However, the previously reported bimetallic sulfides do not have plasmon resonance near-infrared absorption properties. The main reason is that these compounds do not have defective structures. The Cu_3_BiS_3_ nanocrystals designed and synthesized by Hu et al. used these materials for the first time in the diagnosis and treatment of cancer (Ai et al., 2011). By adjusting the copper defects of Cu_3_BiS_3_ nanocrystals, the as-prepared Cu_3_BiS_3_ nanocrystals can simultaneously exhibit intense NIR absorption properties like Cu_2–x_S nanocrystals due to copper defects, and also possess CT imaging capability due to the inherently high X-ray attenuation coefficient of bismuth. However, the size of Cu_3_BiS_3_ nanocrystals is difficult to adjust. The resulting nanocrystals are large in size and have poor photothermal effects (Hessel et al., 2011; Li et al., 2017). Thus the nanocrystals are easily restricted to the kidneys after being injected into the mice via the tail vein. Reducing its size can effectively improve its photothermal performance, and shorten the time in the body (Liu et al., 2016). It has been reported that adjusting the morphological structure or size of materials can improve the MRI/CT imaging performance (Ai et al., 2011; Lee et al., 2012). Therefore, the key to solving the unsatisfactory photothermal/imaging performance of bimetallic sulfide lies in the fine-tuning synthesis of the defect degree, morphology and particle size of bimetallic copper-based sulfide nanomaterials.

In this work, we designed and prepared Cu_3_BiS_3_ nanocrystals with a size of about 12 nm by a modified hydrothermal method in the presence of ethylenediamine. Cu_3_BiS_3_ nanocrystals showed intense absorption in the NIR region, thus exhibited excellent photothermal performance. The photothermal conversion efficiency of Cu_3_BiS_3_ nanocrystals reached up to 58.6% under the irradiation of an 808 nm laser at a power density of 0.4 W cm^–2^. What’s more, Cu_3_BiS_3_ nanocrystals can efficiently kill the macrophages both *in vitro* and *in vivo*, which plays an important role in the development of atherosclerosis, thus can be used as an effective way to inhibit the occurrence of hypertension. In addition, Cu_3_BiS_3_ nanocrystals can be used as an efficient CT contrast agent for carotid inflammation. As far as we know, this is first work on Cu_3_BiS_3_ nanocrystals for CT imaging guided photothermal therapy of arterial inflammation.

## Materials and Methods

### Synthesis of Cu_3_BiS_3_ Nanocrystals

Cu(NO_3_)_2_ (1 mmol), Bi(NO_3_)_3_ (1 mmol), and Sodium dimethyldithiocarbamate (3 mmol) and polyvinylpyrrolidone (PVP, 100 mg) were fully dissolved in deionized (DI, 40 mL) water under stirring, ethylenediamine (100 μL) was then added. The precursor solution was then transferred to a PTFE hydrothermal reactor, and kept at 200°C for 24 h. Black products could be obtained by centrifuge. The products were washed with ethanol and deionized water three times.

### Characterization

Transmission electron microscope was used to detect the shape and size of Cu_3_BiS_3_ nanocrystals. UV-vis spectrophotometer was used to detect the absorption spectrum of Cu_3_BiS_3_ nanocrystals. X-ray photoelectron spectrometer was used to analyze the electronic spectrum of Cu_3_BiS_3_ nanocrystals. X-ray diffractometer was used to detect the phase of Cu_3_BiS_3_ nanocrystals. Inductively coupled plasma emission spectrometer was used to test the concentration of released ions. 808 nm lasers were used as the light source.

### Cell Culture

Raw264.7 macrophage cells was routinely digested and centrifuged. After removing the supernatant, DMEM high glucose complete medium was added to resuspend the cells. The resuspended cells were incubated in a petri dish at a density of 1 × 10^5^/cm^2^ and continue culturing in an incubator (37°C, 5% CO_2_). The cells were digested with trypsin, and continued to expand when the degree of cell fusion reaches 80%.

### CCK-8 Cell Viability Test

Raw264.7 cells were incubated in a 96-well plate. After the cell fusion reached 80%, cells were incubated for 12 h with Cu_3_BiS_3_ nanocrystals with different concentrations (0, 10, 20, 40, 80, 160, 320 ppm). Then the Raw264.7 cells were excited by an 808nm laser (0.3 W/cm^2^, 5 min). Then the medium was removed and the cells were washed with PBS three times to prepare CCK-8 working solution (the ratio of CCK-8 reagent to medium is 1:10). After that, CCK-8 working solution (100 μL) was added to each well. After 1 h, a multi-functional microplate reader was used to detect the absorbance at 450nm wavelength, and the analysis data was collected.

### Live/Dead Cell Staining

The cultured Raw264.7 cells were collected and inoculated in a 96-well plate and in an incubator (37°C, 5% CO_2_). When the degree of cell fusion reached 80%, the cells were incubated with or without Cu_3_BiS_3_ nanocrystals in high glucose medium for 12 h. The cells were divided into different groups: blank control group (Control); Cu_3_BiS_3_ nanocrystals combined with 808 nm laser at different power density (0.1, 0.2, 0.4 W/cm^2^). After the treatments, the culture supernatant was removed, washed with PBS for three times. CalceinAM and PI were then added, and incubated in a 37°C incubator for 20 min. Then the cells were observed under an inverted fluorescence microscope.

### Animal Model Construction

All animal experiments were approved by the Animal Ethics Committee of The First Affiliated Hospital of Bengbu Medical College. 28 of 8-week-old ApoE-/- mice were selected and fixed on the rat board after anesthetized with chloral hydrate. The left carotid artery was exposed under the microscope via making a longitudinal incision in the left neck. A 5 mm length silicone tube was placed around the left carotid artery to wrap it around the left carotid artery. The silk thread was ligated and fixed, and the skin and subcutaneous layers were sutured layer by layer, placed in a 35°C incubator to wake up, and then put back into the cage.

### Infrared Thermal Imaging and Photothermal Therapy *in vivo*

Two weeks after the surgery, ApoE-/- mice were divided into two groups: control group and experiment group. The mice were locally injected with PBS or Cu_3_BiS_3_ nanocrystals. The mice were simultaneously irradiated to the 808 nm lasers (0.4 W/cm^2^, 5 min). An infrared thermal imaging camera was used to detect the temperature change of the mice during the treatment.

## Histological Analysis

The overall status of the mice in each group was observed after surgery. The mice were sacrificed by spinal dislocation on 14 days of photothermal treatment. The left carotid artery and main organs of each group were surgically obtained and dehydrated with 10% sucrose for 1.5–2 h. After that, they are transferred to 30% sucrose for soaking overnight, and then embed frozen sections with OCT to make continuous sections with a thickness of 6–8 μm. The slices were dried overnight in a dark and ventilated place. The next day, the slices were loaded into a slice box and sealed and stored in a −20° refrigerator; or after dehydration, they were embedded into paraffin blocks to make continuous slices with a thickness of 6–8 μm. When necessary, the sections were stained for immunofluorescence or immunohistochemical, observed under a fluorescence microscope.

### Carotid Tissue Immunofluorescence Staining

Carotid artery slices were soaked in PBS for three times (5 min each time) to remove OCT embedding agent. The membrane was perforated with 0.1% Triton-X for 10 min, rinsed with PBS for three times. 5% goat serum was blocked at room temperature for 30 min. Diluted anti-mouse CD68 and CD31 antibodies were added, respectively. The slides were removed the next day and rinsed with PBS for three times. DAPI (1:500) was added dropwise to stain nuclei for 30 s, rinsed twice with PBS for 5 min each time. After anti-fluorescence quenching, the tablet was sealed, observed and photographed under a fluorescence microscope.

### Carotid Artery Hematoxylin-Eosin Staining

The slices of carotid artery and main organ tissues were rinsed with distilled water for three times, 5 min each time. Then the slices were stained with hematoxylin and eosin (HE) dye for 2 min, rinsed with distilled water. The slices were gradually dehydrated with 70, 85, 95, and 100% alcohol in sequence. Finally, The slices were observed under microscope after sealing with neutral gum.

### CT Imaging

Cu_3_BiS_3_ nanocrystals with varied concentrations (0, 0.5, 1.0, 2.0, 4.0 mg/mL) were placed in PE tubes and then scanned by CT imaging system. CT imaging *in vivo* was performed using Cu_3_BiS_3_ nanocrystals as CT contrast agents. ApoE-/- mice with carotid inflammation model were locally injected with Cu_3_BiS_3_ nanocrystals dispersed in PBS in surgical site. Pre- and post-injection, the mice were scanned by the same CT imaging system.

### Long-Term Toxicity *in vivo*

The long-term toxicity *in vivo* of Cu_3_BiS_3_ nanocrystals was evaluated by H&E analysis of major organs and the biodistribution of Cu_3_BiS_3_ nanocrystals in main organs. For H&E analysis of major organs (lung, liver, spleen, kidney, and heart), healthy mice were intravenously injected with Cu_3_BiS_3_ nanocrystals (10 mg⋅kg^–1^, treatment group) or PBS solution (control group), major organs were collected for H&E analysis. The biodistribution of the Cu_3_BiS_3_ nanocrystals was evaluated by intravenous injection with Cu_3_BiS_3_ nanocrystals (10 mg⋅kg^–1^). Major organs were achieved at different time points (i.e., 1, 3, 6, 9 days) for H&E analysis.

## Results and Discussion

**FIGURE 1:**
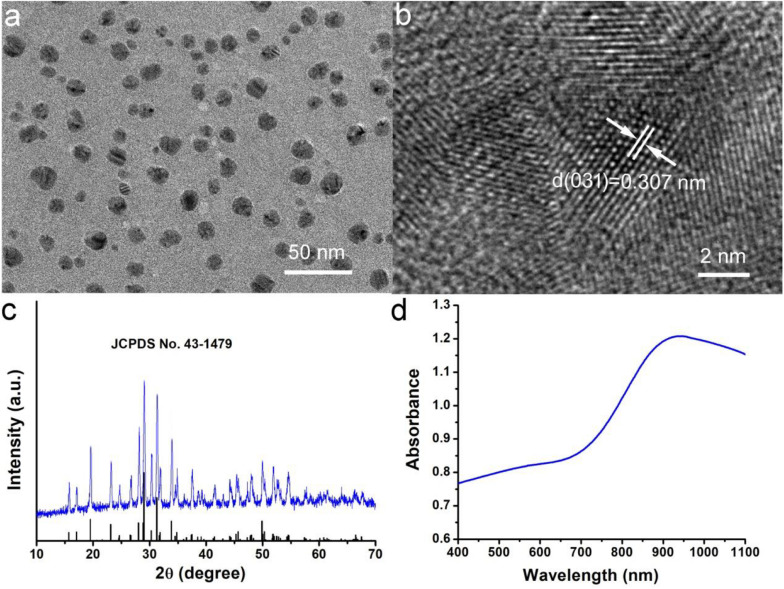
**(a)** Typical TEM image of Cu_3_BiS_3_ nanocrystals. **(b)** High resolution TEM image of Cu_3_BiS_3_ nanocrystals. **(c)** XRD pattern of Cu_3_BiS_3_ nanocrystals. **(d)** UV-vis-NIR spectra of Cu_3_BiS_3_ nanocrystals at room temperature.

Hydrophilic Cu_3_BiS_3_ nanocrystals were synthesized by hydrothermal method. In Figure 1a, transmission electron microscopy (TEM) image presented that the synthesized Cu_3_BiS_3_ nanocrystals showed good dispersion. The size of nanocrystals was about 12 nm (Supplementary Figure S1) which was much smaller than that of previously reported Cu_3_BiS_3_ nanocrystals (Li et al., 2017), indicating that the synthesized nanocrystals were more suitable for bioapplication. This reduction in particle size may be related to the presence of ethylenediamine. As an ion complexing agent, ethylenediamine can control the release rate of iron ions in the reaction, and the size of nanocrystals can be reduced (Liu et al., 2020). We further studied the microstructure of Cu_3_BiS_3_ nanocrystals with high-resolution transmission electron microscopy (HRTEM). As shown in Figure 1b, the single crystal plane spacing was 0.307 nm, which corresponded to the crystal plane spacing of the (031) plane of Cu_3_BiS_3_ nanocrystals. Additionally, all the X-ray diffraction patterns (XRD, Figure 1c) of Cu_3_BiS_3_ nanocrystals can be well indexed as Cu_3_BiS_3_ nanocrystals. The lattice parameters were consistent with those on the JCPDS file (No. 16-0713). The perfect match indicated that the synthesized Cu_3_BiS_3_ nanocrystals with high crystallinity and high purity. We then measured the optical property. As expected, the Cu_3_BiS_3_ nanocrystals showed strong absorption in NIR region centered at 905 nm (Figure 1D), indicating that Cu_3_BiS_3_ nanocrystals showed great potential as photothermal agents.

**FIGURE 2:**
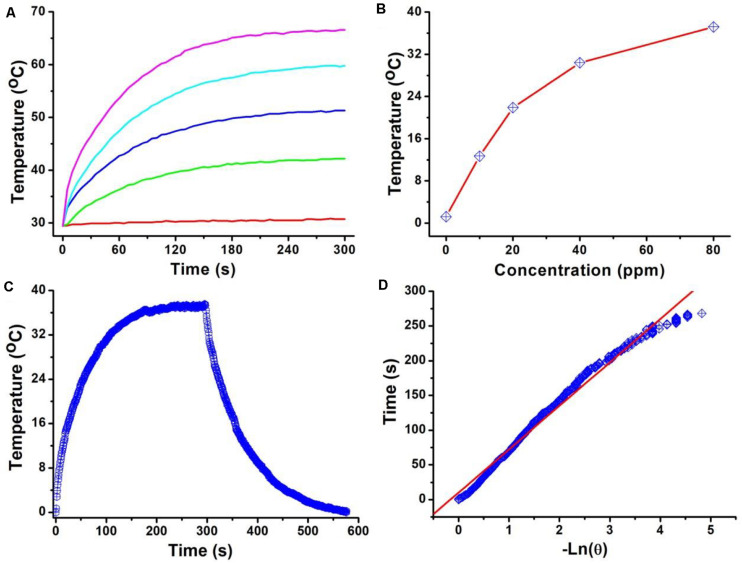
**(A)** Temperature of Cu_3_BiS_3_ nanocrystals with different concentrations (0, 20, 40, 60, 80 ppm) under the excitation of an 808 nm laser (0.4 W cm^– 2^, 300 s). **(B)** Temperature change as a function of the concentration of Cu_3_BiS_3_ nanocrystals. **(C)** Temperature change of Cu_3_BiS_3_ nanocrystals (80 ppm) under the irradiation of the 808 nm laser (0.4 W cm^– 2^, 300 s) and shutting off the laser to cool to the room temperature. **(D)** Time constant of Cu_3_BiS_3_ nanocrystals from the cooling process.

The intense NIR absorption of Cu_3_BiS_3_ nanocrystals motivated us to evaluate the photothermal performance of Cu_3_BiS_3_ nanocrystals. The aqueous dispersions of nanocrystals with varied concentrations were placed in the PE tubes, and excited by an 808 nm laser. The temperature change was recorded by infrared thermal imager. As shown in Figure 2A, the temperature of aqueous dispersions of nanocrystals increased dramatically under the irradiation of the 808 nm laser, while the temperature of pure water showed little change, indicating that Cu_3_BiS_3_ nanocrystals exhibited excellent photothermal effect. As the concentration increased, the elevated temperature increased. Obviously, Cu_3_BiS_3_ nanocrystals showed concentration-dependent photothermal effect. Figure 2B provides the direct relationship between the concentration of Cu_3_BiS_3_ nanocrystals and the temperature. When the concentration was 80 ppm, the temperature of the aqueous dispersion was increased by 37.2°C, while the temperature of pure water was increased by less than 2°C, demonstrating the excellent photothermal performance of Cu_3_BiS_3_ nanocrystals. To further evaluate the photothermal performance of Cu_3_BiS_3_ nanocrystals, the photothermal conversion efficiency of Cu_3_BiS_3_ nanocrystals was measured according to previously reported methods. As shown in Figure 2C, 80 ppm of Cu_3_BiS_3_ nanocrystals was excited by the 808 nm laser (0.3 W) until the temperature reached equilibrium and no longer changed. Then the laser was removed away, and the cooling temperature during the cooling process was recorded (Figure 2D). The time constant can be calculated to be 98.3 s. Thus, the photothermal conversion efficiency of Cu_3_BiS_3_ was calculated to be 58.6% which is high enough for photothermal therapy.

In order to detect the cytotoxicity of Cu_3_BiS_3_ nanocrystals, we used different concentrations of Cu_3_BiS_3_ nanocrystals (0∼320 ppm) to co-culture with macrophages for 12 h, and then tested the cell activity of each group by CCK-8 experiment. The results showed that the concentration was below 160 ppm, Cu_3_BiS_3_ nanocrystals had no significant effect on the activity of macrophages (Raw264.7); when the concentration reached 320 ppm, Raw264.7 activity decreased significantly (see Supplementary Figure S2A). Based on the photothermal curves of Cu_3_BiS_3_ nanocrystals under the action of near-infrared light, Cu_3_BiS_3_ nanocrystals showed a good heating effect at a concentration of 40 ppm; the temperature was increase by 20°C. We thus chose 40 ppm as the concentration used in subsequent experiments.

**FIGURE 3:**
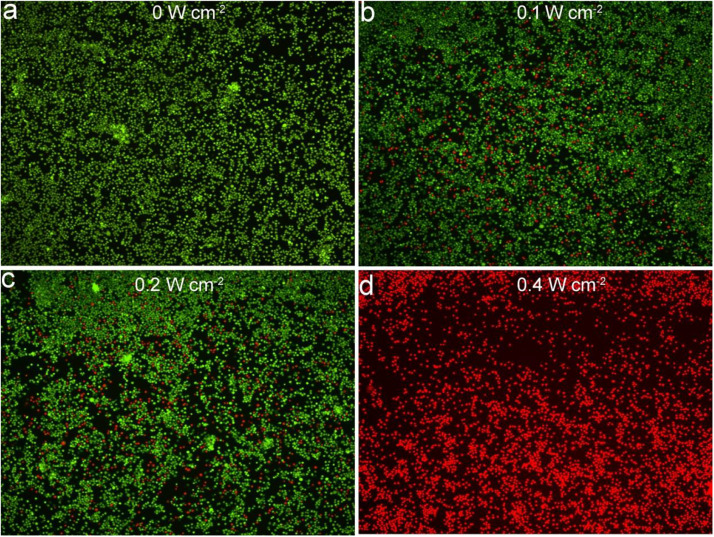
Confocal images of fluorescence staining live/dead cells incubated with Cu_3_BiS_3_ nanocrystals then excited by an 808 nm laser with varied power densities: **(a)** 0 W cm^– 2^, **(b)** 0.1 W cm^– 2^, **(c)** 0.2 W cm^– 2^, **(d)** 0.4 W cm^– 2^. Magnification: 200 times.

Macrophage infiltration is one of the main causes of arterial inflammation and arterial stenosis, so we examined the effect of thermal effects based on Cu_3_BiS_3_ nanocrystals on macrophage activity. Cell phagocytosis experiments without using an additional target or selective groups have been studied by many groups (Van Furth et al., 1979; Han et al., 2016; Wang et al., 2019). Nanoparticles were mostly aggregated in lysosomes, and macrophages have much larger amounts of lysosomes than endothelial cells (Van Furth et al., 1979; Han et al., 2016; Wang et al., 2019). In that case, macrophages, as a kind immunocyte, phagocytized a higher proportion of nanoparticles than other cells. As shown in Supplementary Figure S2B, the CCK-8 test found that most cells under the co-action of Cu_3_BiS_3_ nanocrystals and 808 nm laser irradiation (0.4 W cm^–2^), and the cell activity was significantly higher than those of cells treated with NIR laser at lower power density. As expected, the cell activity of the control group (no NIR laser irradiation) did not change significantly. Meanwhile, NIR Laser alone had almost no effect on the cell activity (Supplementary Figure S3). In addition, we also stained the living/dead cells of each treatment group to further clarify the status of the cells in each treatment group. As shown in Figures 3a–d, no significant cell death was observed in the control group, while nearly 40% of the cells treated with the 808 nm laser (0.1 W cm^–2^) were killed, 55% of the cells treated with the 808 nm laser with a higher power density of 0.2 W cm^–2^ were killed, and more than 90% of the cells treated with the 808 nm laser were killed when the laser power density was 0.4 W cm^–2^, which was consistent with the CCK-8 results.

**FIGURE 4:**
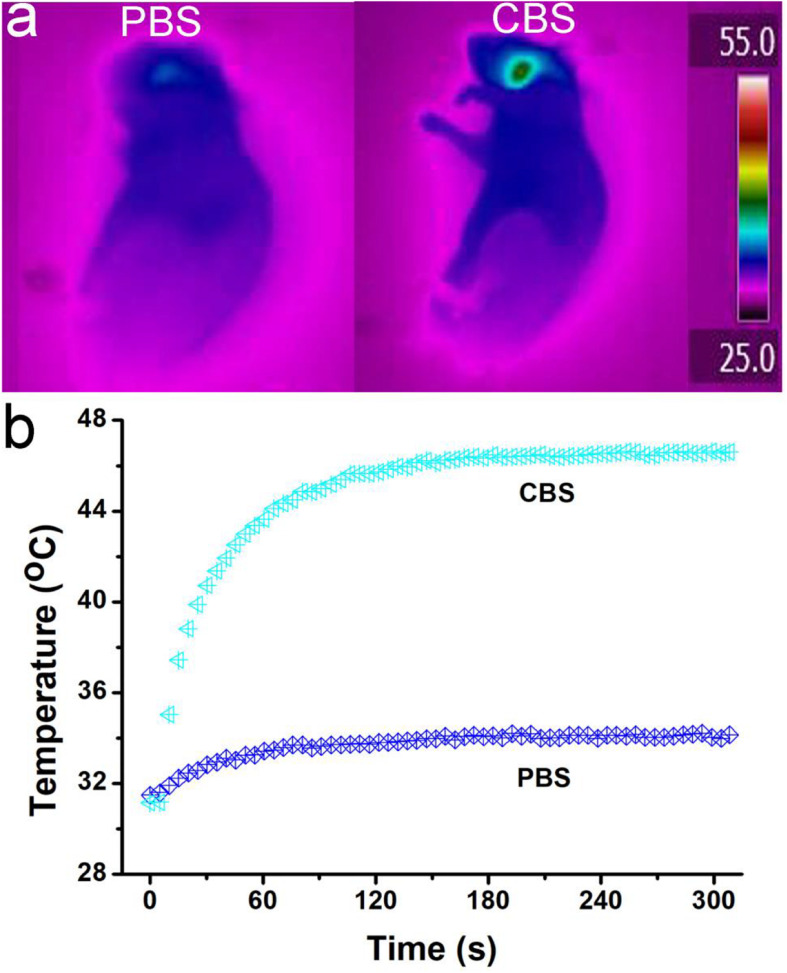
**(a)** Infrared thermography of ApoE-/- mice treated by injection of PBS (left)/Cu_3_BiS_3_ (CBS, right) nanocrystals (right) and the 808 nm laser irradiation (0.4 W cm^– 2^) for 300 s. **(b)** Temperature change during the photothermal therapy.

After verifying that Cu_3_BiS_3_ nanocrystals can effectively kill macrophages *in vitro*, we further used ApoE-/- mice to make arterial inflammation and stenosis models for photothermal therapy *in vivo*. ApoE-/- mice were divided into two groups: control group and experiment group. The mice were locally injected with PBS or Cu_3_BiS_3_ nanocrystals. The mice were simultaneously excited by the 808 nm lasers (0.4 W/cm^2^, 5 min). An infrared thermal imaging camera was used to detect the surface temperature change of the mice during the treatment. The infrared thermal imager dynamically recorded the local temperature changes of the left neck of the mouse. As shown in Figures 4a,b, the local temperature of the Cu_3_BiS_3_ + PTT group can rapidly increase to 46.6°C within 300 s, while the local temperature of the PBS + PTT group still kept below 35°C during the whole process, showing an obvious contrast in infrared thermography. Therefore, Cu_3_BiS_3_ nanocrystals still showed excellent photothermal effect *in vivo* driven by the 808 nm laser.

**FIGURE 5:**
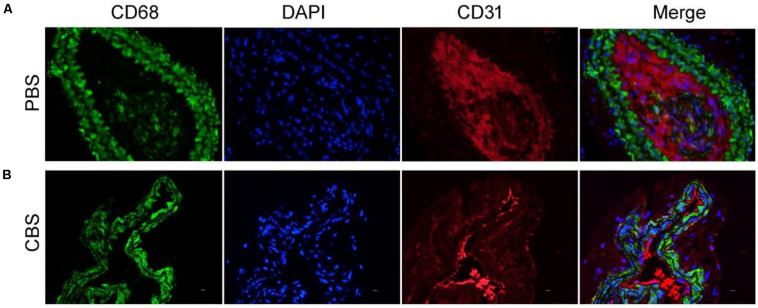
Immunofluorescence staining of macrophages, endothelial cells, and merge pictures in the **(A)** control group (PBS) and **(B)** experimental group (CBS). Magnification: 200 times.

Two weeks after photothermal therapy *in vivo*, the left carotid artery of each group of mice was removed for immunofluorescence staining. In immunofluorescence, we used CD68 as a marker for macrophages and CD31 as a marker for vascular smooth muscle cells. The results showed that the number of infiltrated CD68^+^ macrophages in the middle artery wall of the control group (PBS + NIR, Figure 5A) was much higher than that of experiment (Cu_3_BiS_3_ + PTT, Figure 5B) group. This indicates that the photothermal therapy based on Cu_3_BiS_3_ nanocrystals can effectively inhibit the infiltration of macrophages in the inflammatory arterial wall in a short period of time, which may reduce the adverse results caused by the infiltration of a large number of inflammatory macrophages.

**FIGURE 6:**
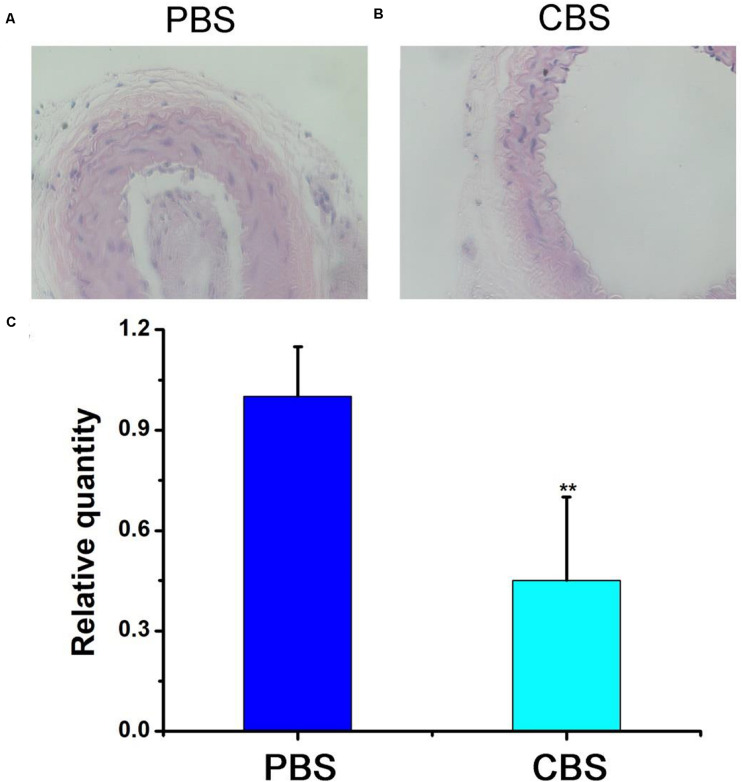
HE staining of the carotid artery in the **(A)** control group (PBS) and **(B)** experimental group (CBS). **(C)** Statistics. Magnification: 200 times.

In order to further evaluate the effect of ablation of arterial wall macrophages on reducing the thickness of the arterial wall and inhibiting the progress of arterial stenosis, we tested the thickness of the carotid artery wall of mice by HE staining. The results showed that the thickness of the intima/media in the experimental (CBS + NIR) group was much lower than that in the control (PBS + NIR) group (Figures 6A,B). Effectively inhibit the thickening of the intima/media of the arterial wall, thereby reducing the occurrence of arterial stenosis. In addition, this result was consistent with the arterial wall inflammatory macrophage infiltration results (Figure 6C). The relative quantity of macrophages in the experimental group was much lower than that in the control group. The arterial intima/media thickness was positively correlated with the amount of arterial wall macrophage infiltration to a certain extent, further indicating that inflammatory macrophage infiltration in the artery, the key role in wall hyperplasia. The most sensitive part in a carotid artery should be the thin layer of endothelial cells, however, in the previous study, it has found macrophages are even more vulnerable than endothelial cells (Peng et al., 2015, 2020; Wang et al., 2019). And because the highest temperature in this photothermal proves is lower than 50°C (Figure 4b), it would not cause much damage to the carotid artery. According to previous studies, photothermal process can cause some inflammation response, while there is also reports indicate that the laser irradiation can inhibited excessive inflammation, facilitated angiogenesis, as well as improving revascularization (Dong et al., 2018; Ma et al., 2018). As for the material itself, previous work has been proofed that they have little inflammation response (Peng et al., 2020). And nti-CD68 receptor-targeted Fe-doped hollow silica nanoparticles were used as dual-modal US/MRI contrast agent for identifying macrophages of aorta ventralis atherosclerotic plaques in ApoE-/- mice (Ji et al., 2018). To sum up, we can think that photothermal therapy based on Cu_3_BiS_3_ nanocrystals can effectively suppress the thickening of the arterial wall by ablating inflammatory macrophages in the arterial wall in the short term, thereby effectively inhibiting the occurrence of arterial stenosis.

**FIGURE 7:**
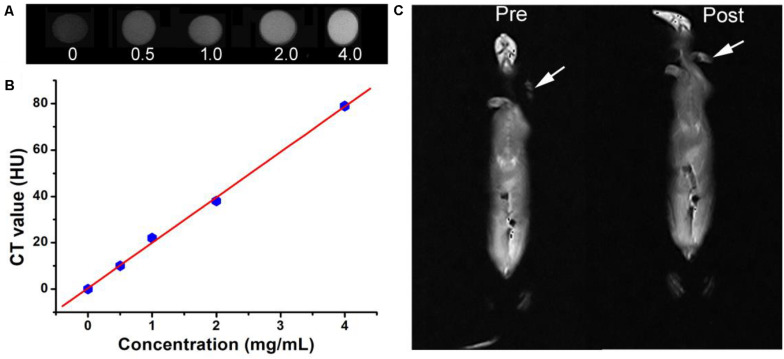
**(A)** CT image *in vitro* of Cu_3_BiS_3_ nanocrystals with varied concentrations (0, 0.5, 1.0, 2.0, 4.0 mg/mL). **(B)** CT value as a function of the concentration of Cu_3_BiS_3_ nanocrystals. **(C)** CT images *in vivo* of the ApoE-/- mice pre- and post-injected with Cu_3_BiS_3_ nanocrystals (4 mg/mL).

In addition to the excellent photothermal effect, Cu_3_BiS_3_ nanocrystals also exhibited excellent CT imaging performance because of the high attenuation coefficient of bismuth. Cu_3_BiS_3_ nanocrystals with varied concentrations (0, 0.5, 1.0, 2.0, 4.0 mg/mL) were placed in PE tubes and then scanned by CT imaging system. As shown in Figure 7A, CT signal was increased with the increase of the concentration of Cu_3_BiS_3_ nanocrystals; the CT signal of Cu_3_BiS_3_ nanocrystals with a concentration of 4 mg/mL was higher than that of pure water, indicating the excellent CT imaging performance. In addition, CT values increased linearly with the concentration of Cu_3_BiS_3_ nanocrystals. The slope was calculated to be 19.55 HU L/g, higher than some of previously reported CT contrast agents (Figure 7B). We then studied the CT imaging *in vivo* using Cu_3_BiS_3_ nanocrystals as CT contrast agents. ApoE-/- mice with carotid inflammation model were locally injected with Cu_3_BiS_3_ nanocrystals dispersed in PBS in surgical site. Pre- and post-injection, the mice were scanned by a CT imaging system. As shown in Figure 7C, there was a significant difference in CT signal pre- and post- injection of Cu_3_BiS_3_ nanocrystals in the artery of ApoE-/- mice. Therefore, Cu_3_BiS_3_ nanocrystals can be an efficient CT contrast agent for CT imaging of carotid inflammation.

The long-term toxicity *in vivo* of Cu_3_BiS_3_ nanocrystals was evaluated by H&E analysis of major organs and the biodistribution of Cu_3_BiS_3_ nanocrystals in main organs. For H&E analysis of major organs (lung, liver, spleen, kidney, and heart), healthy mice were intravenously injected with Cu_3_BiS_3_ nanocrystals (10 mg⋅kg^–1^, treatment group) or PBS solution (control group), major organs were collected for H&E analysis. It was found that the shape and size of the cells both in the treatment group and in the control group almost showed no change (Supplementary Figure S4), indicating the low toxicity *in vivo* of Cu_3_BiS_3_ nanocrystals. The biodistribution of the Cu_3_BiS_3_ nanocrystals was evaluated by intravenous injection with Cu_3_BiS_3_ nanocrystals (10 mg kg^–1^). Major organs were achieved at different time points (i.e., 1, 3, 6, 9 days). It showed (Supplementary Figure S5) that the Cu_3_BiS_3_ nanocrystals mainly accumulate in the kidney and spleen after the intravenous administration. The content in these two organs gradually decreased over time, indicating that Cu_3_BiS_3_ nanocrystals were mainly degraded through these two organs.

## Conclusion

In conclusion, Cu_3_BiS_3_ nanocrystals with a size of about 12 nm were successfully designed and prepared by a simple hydrothermal method. Cu_3_BiS_3_ nanocrystals showed excellent photothermal effect when driven by an 808 nm laser. The photothermal conversion efficiency of Cu_3_BiS_3_ nanocrystals was calculated to be 58.6% due to the strong NIR absorption. Under the irradiation of an 808 nm laser, Cu_3_BiS_3_ nanocrystals can efficiently kill the macrophages both *in vitro* and *in vivo*, which plays an important role in the development of atherosclerosis, thus can be used as an effective way to inhibit the occurrence of hypertension. In addition, Cu_3_BiS_3_ nanocrystals can be used as an efficient CT contrast agent for carotid inflammation. Therefore, Cu_3_BiS_3_ nanocrystals show great potential for CT imaging guided photothermal therapy of arterial inflammation.

## Data Availability Statement

All datasets generated for this study are included in the article/Supplementary Material.

## Ethics Statement

The animal study was reviewed and approved by the First Affiliated Hospital of Bengbu Medical College.

## Author Contributions

RL, JZ, and YG designed the project and performed the experimental data analysis. RL, JZ, CY, and ZN carried out the experiment. RL and YG wrote the manuscript. All the authors contributed to discussion of the results.

## Conflict of Interest

The authors declare that the research was conducted in the absence of any commercial or financial relationships that could be construed as a potential conflict of interest.

## References

[B1] Ai K. Liu Y. Liu J. Yuan Q. He Y. Lu L. (2011). Large-scale synthesis of bi2s3nanodots as a contrast agent for in Vivo X-ray computed tomography imaging. *Adv. Mater.* 23 4886–4891. 10.1002/adma.201103289 21956662

[B2] Chen Z. Wang Q. Wang H. Zhang L. Song G. Song L. (2013). Ultrathin PEGylated W18O49Nanowires as a New 980 nm-laser-driven photothermal agent for efficient ablation of cancer cells in vivo. *Adv. Mater.* 25 2095–2100. 10.1021/acsami.9b12258 23427112

[B3] Dong Q. Wang X. W. Hu X. X. Xiao L. Q. Zhang L. Song L. J. (2018). Simultaneous application of photothermal therapy and an anti-inflammatory prodrug using pyrene-aspirin-loaded gold nanorod graphitic nanocapsules. *Angew. Chem. Intern. Edn.* 57 177–181. 10.1002/anie.201709648 29125675

[B4] Han C. Z. Juncadella I. J. Kinchen J. M. Buckley M. W. Klibanov A. L. Dryden K. (2016). Macrophages redirect phagocytosis by non-professional phagocytes and influence inflammation. *Nature* 539 570–574. 10.1038/nature20141 27820945 PMC5799085

[B5] Hessel C. M. Pattani V. P. Rasch M. Panthani M. G. Koo B. Tunnell J. W. (2011). Copper selenide nanocrystals for photothermal therapy. *Nano Lett*. 11 2560–2566. 10.1021/nl201400z 21553924 PMC3111000

[B6] Ji R. Li X. Y. Zhou C. Tian Q. W. Li C. Xia S. J. (2018). Identifying macrophage enrichment in atherosclerotic plaques by targeting dual-modal US imaging/MRI based on biodegradable Fe-doped hollow silica nanospheres conjugated with anti-CD68 antibody. *Nanoscale* 10 20246–20255. 10.1039/c8nr04703k 30361722

[B7] Jin Q. Liu J. Zhu W. Dong Z. Liu Z. Cheng L. (2018). Albumin-assisted synthesis of ultrasmall FeS2 nanodots for imaging-guided photothermal enhanced photodynamic therapy. *ACS Appl. Mater. Interf.* 10 332–340. 10.1021/acsami.7b16890 29220162

[B8] Lee D.-E. Koo H. Sun I.-C. Ryu J. H. Kim K. Kwon I. C. (2012). Multifunctional nanoparticles for multimodal imaging and theragnosis. *Chem. Soc. Rev.* 41 2656–2672. 10.1039/c2cs15261d 22189429

[B9] Li B. Wang Q. Zou R. Liu X. Xu K. Li W. (2014). Cu7.2*S*4 nanocrystals: a novel photothermal agent with a 56.7% photothermal conversion efficiency for photothermal therapy of cancer cells. *Nanoscale* 6 3274–3282. 10.1039/c3nr06242b 24509646

[B10] Li B. Yuan F. He G. Han X. Wang X. Qin J. (2017). Ultrasmall CuCo2S4Nanocrystals: all-in-one theragnosis nanoplatform with magnetic resonance/near-infrared imaging for efficiently photothermal therapy of tumors. *Adv. Funct. Mater.* 27:1606218 10.1002/adfm.201606218

[B11] Li J. Pu K. (2019). Development of organic semiconducting materials for deep-tissue optical imaging, phototherapy and photoactivation. *Chem. Soc. Rev.* 48 38–71. 10.1039/c8cs00001h 30387803

[B12] Li J. C. Rao J. H. Pu K. Y. (2018). Recent progress on semiconducting polymer nanoparticles for molecular imaging and cancer phototherapy. *Biomaterials* 155 217–235. 10.1016/j.biomaterials.2017.11.025 29190479 PMC5978728

[B13] Li W. Zamani R. Rivera Gil P. Pelaz B. Ibanez M. Cadavid D. (2013). CuTe nanocrystals: shape and size control, plasmonic properties, and use as SERS probes and photothermal agents. *J. Am. Chem. Soc.* 135 7098–7101. 10.1021/ja401428e 23647089

[B14] Libby P. Ridker P. M. Hansson G. K. (2011). Progress and challenges in translating the biology of atherosclerosis. *Nature* 473 317–325. 10.1038/nature10146 21593864

[B15] Liu J. Guo X. Zhao Z. Li B. Qin J. Peng Z. (2020). Fe3S4 nanoparticles for arterial inflammation therapy: integration of magnetic hyperthermia and photothermal treatment. *Appl. Mater. Today* 18:100457 10.1016/j.apmt.2019.100457

[B16] Liu J. Wang P. Zhang X. Wang L. Wang D. Gu Z. (2016). Rapid degradation and high renal clearance of Cu3BiS3 nanodots for efficient cancer diagnosis and photothermal therapy in vivo. *ACS Nano* 10 4587–4598. 10.1021/acsnano.6b00745 27014806

[B17] Ma J. X. Yang Q. M. Xia Y. C. Zhang W. G. Nie F. F. (2018). Effect of 810nm near-infrared laser on revascularization of ischemic flaps in rats. *Photomed. Laser Surg.* 36 290–297. 10.1089/pho.2017.4360 29882737

[B18] Meng Z. Wei F. Ma W. Yu N. Wei P. Wang Z. (2016). Design and synthesis of “All-in-One” multifunctional FeS2 nanoparticles for magnetic resonance and near-infrared imaging guided photothermal therapy of tumors. *Adv. Funct. Mater.* 26 8231–8242. 10.1002/adfm.201603776

[B19] Ni D. Zhang J. Wang J. Hu P. Jin Y. Tang Z. (2017). Oxygen vacancy enables markedly enhanced magnetic resonance imaging-guided photothermal therapy of a Gd(3+)-doped contrast agent. *ACS Nano* 11 4256–4264. 10.1021/acsnano.7b01297 28323405

[B20] Peng X. Liu J. C. Ming C. Li B. Zhao Z. Ye K. C. (2020). AgFeS2 nanoparticles as a novel photothermal platform for effective artery stenosis therapy. *Nanoscale* 12 11288–11296. 10.1039/d0nr01587c 32420577

[B21] Peng Z. Qin J. Li B. Ye K. Zhang Y. Yang X. (2015). An effective approach to reduce inflammation and stenosis in carotid artery: polypyrrole nanoparticle-based photothermal therapy. *Nanoscale* 7 7682–7691. 10.1039/c5nr00542f 25833402

[B22] Qin J. Peng Z. Li B. Ye K. Zhang Y. Yuan F. (2015). Gold nanorods as a theranostic platform for in vitro and in vivo imaging and photothermal therapy of inflammatory macrophages. *Nanoscale* 7 13991–14001. 10.1039/c5nr02521d 26228112

[B23] Robinson J. T. Tabakman S. M. Liang Y. Wang H. Casalongue H. S. Vinh D. (2011). Ultrasmall reduced graphene oxide with high near-infrared absorbance for photothermal therapy. *J. Am. Chem. Soc.* 133 6825–6831. 10.1021/ja2010175 21476500

[B24] Robinson J. T. Welsher K. Tabakman S. M. Sherlock S. P. Wang H. Luong R. (2010). High performance in vivo near-IR (>1 mum) imaging and photothermal cancer therapy with carbon nanotubes. *Nano Res.* 3 779–793. 10.1007/s12274-010-0045-1 21804931 PMC3143483

[B25] Shao J. Xie H. Huang H. Li Z. Sun Z. Xu Y. (2016). Biodegradable black phosphorus-based nanospheres for in vivo photothermal cancer therapy. *Nat. Commun.* 7:12967. 10.1038/ncomms12967 27686999 PMC5056460

[B26] Song G. S. Ji C. H. Liang C. Song X. J. Yi X. Dong Z. L. (2017). TaOx decorated perfluorocarbon nanodroplets as oxygen reservoirs to overcome tumor hypoxia and enhance cancer radiotherapy. *Biomaterials* 112 257–263. 10.1016/j.biomaterials.2016.10.020 27768978

[B27] Tabas I. (2010). Macrophage death and defective inflammation resolution in atherosclerosis. *Nat. Rev. Immunol.* 10 36–46. 10.1038/nri2675 19960040 PMC2854623

[B28] Tan C. Cao X. Wu X. J. He Q. Yang J. Zhang X. (2017). Recent advances in ultrathin two-dimensional nanomaterials. *Chem. Rev.* 117:6225. 10.1021/acs.chemrev.6b00558 28306244

[B29] Tian Q. Hu J. Zhu Y. Zou R. Chen Z. Yang S. (2013). Sub-10 nm Fe3O4@Cu2-xS core-shell nanoparticles for dual-modal imaging and photothermal therapy. *J. Am. Chem. Soc.* 135 8571–8577. 10.1021/ja4013497 23687972

[B30] Tzoulaki I. Elliott P. Kontis V. Ezzati M. (2016). Worldwide exposures to cardiovascular risk factors and associated health effects: current knowledge and data Gaps. *Circulation* 133 2314–2333. 10.1161/CIRCULATIONAHA.115.008718 27267538

[B31] Van Furth R. Raeburn J. A. Van Zwet T. L. (1979). Characteristics of human mononuclear phagocytes. *Blood* 54 485–500. 10.1182/blood.V54.2.485.485 454850

[B32] Wang X. Wu X. Qin J. Ye K. Lai F. Li B. (2019). Differential phagocytosis-based photothermal ablation of inflammatory macrophages in atherosclerotic disease. *ACS Appl. Mater. Interf.* 11 41009–41018. 10.1021/acsami.9b12258 31599564

[B33] Zhang X. Liu J. C. Yang X. R. He G. J. Li B. Qin J. B. (2019). CuCo2S4 nanocrystals as a nanoplatform for photothermal therapy of arterial inflammation. *Nanoscale* 11 9733–9742. 31066405 10.1039/c9nr00772e

[B34] Zhou M. Tian M. Li C. (2016). Copper-based nanomaterials for cancer imaging and therapy. *Bioconjug. Chem.* 27 1188–1199. 10.1021/acs.bioconjchem.6b00156 27094828

