# Supplementary material for: Cu3BiS3 Nanocrystals as Efficient Nanoplatforms for CT Imaging Guided Photothermal Therapy of Arterial Inflammation
Source: Front Bioeng Biotechnol. 2020 Aug 14;8:981. doi: 10.3389/fbioe.2020.00981 (PMC7457067; doi:10.3389/fbioe.2020.00981)
Supplement: Supplementary file 1 [file Data_Sheet_1.docx]

**Supporting Information**

**Cu_3_BiS_3_ nanocrystals as efficient nanoplatforms for CT imaging guided photothermal therapy of arterial inflammation**

Ran Lu^1^, Jingyi Zhu^2^, Chaowen Yu^1^, Zhonglin Nie^1^, Yong Gao^1^*

^1^ Department of vascular surgery, The First Affiliated Hospital of Bengbu Medical College, Bengbu 233004, China.

^2^ School of Pharmaceutical Sciences, Nanjing Tech University, Nanjing 211816, China.


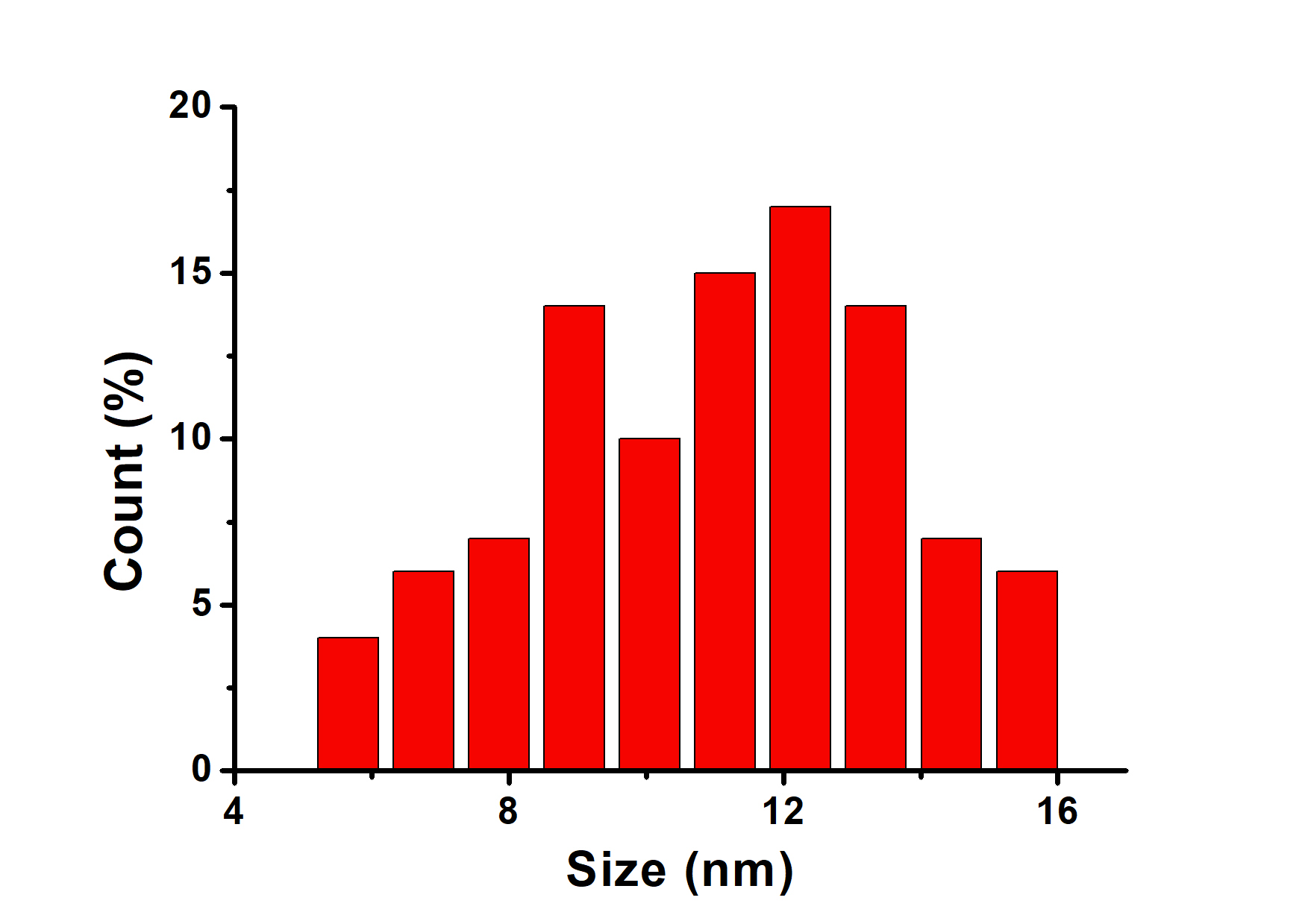


**Figure S1**. Size distribution of Cu_3_BiS_3_ nanocrystals.


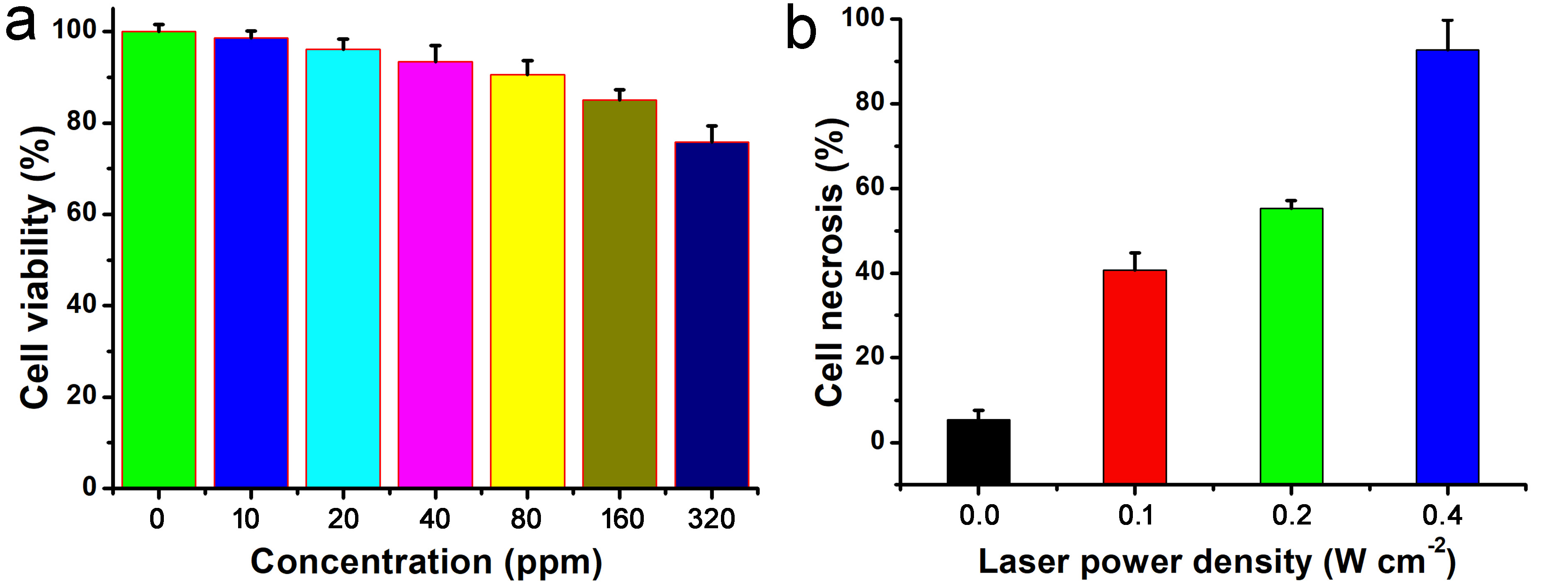


**Figure S2**. (a) Cell viability of macrophages incubated with Cu_3_BiS_3_ nanocrystals at different concentrations. (b) Cell necrosis incubated with Cu_3_BiS_3_ nanocrystals under the irradiation of an 808 nm laser with different power densities.


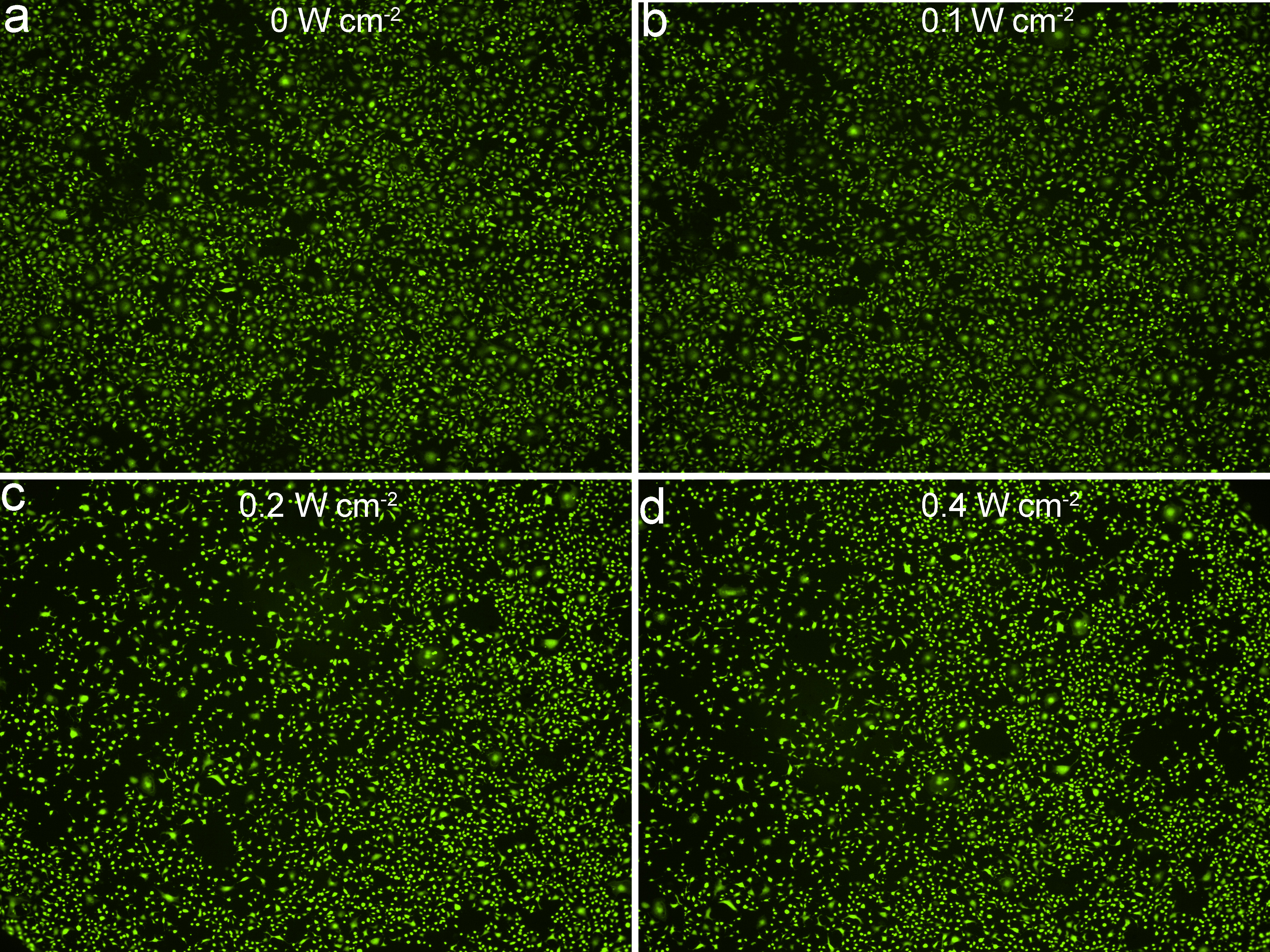


**Figure S3**. Confocal images of fluorescence staining live/dead cells incubated with PBS solution then exicted by an 808 nm laser with varied power densities: (a) 0 W cm^-2^, (b) 0.1 W cm^-2^, (c) 0.2 W cm^-2^, (d) 0.4 W cm^-2^. Magnification: 200 times.


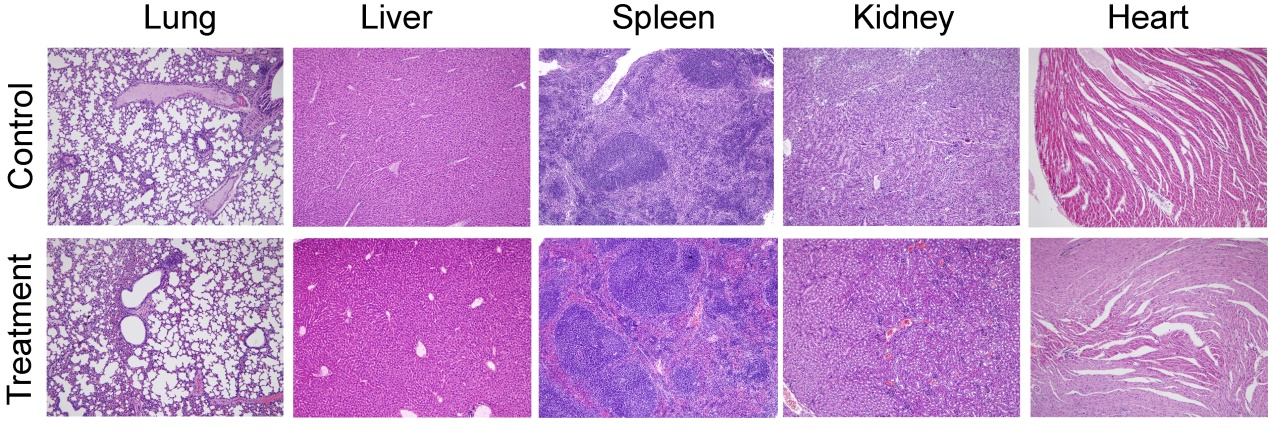


**Figure S4**. The typical images of H&E analysis of major organs from sacrificed mice. Magnification: 100 times.


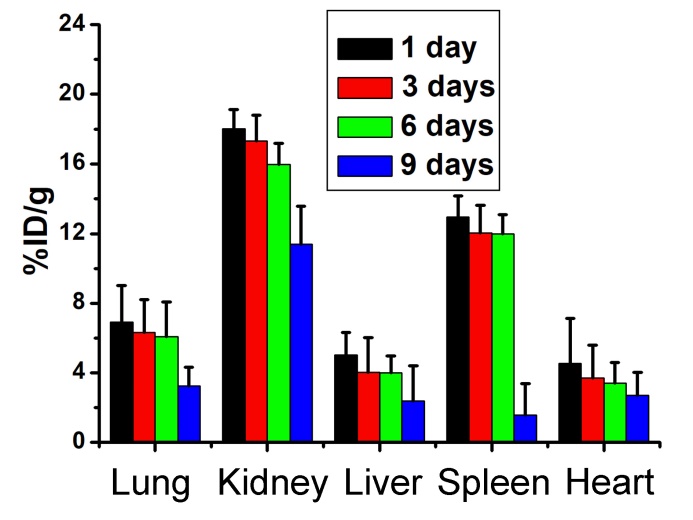


**Figure S5**. Biodistribution of Cu_3_BiS_3_ nanoassemblies in main organs.
